# A comparative study among machine learning and numerical models for simulating groundwater dynamics in the Heihe River Basin, northwestern China

**DOI:** 10.1038/s41598-020-60698-9

**Published:** 2020-03-03

**Authors:** Chong Chen, Wei He, Han Zhou, Yaru Xue, Mingda Zhu

**Affiliations:** 1 China University of Petroleum-Beijing, College of Information Science and Engineering, Beijing, 102249 China; 2 China University of Petroleum-Beijing, State Key Laboratory of Petroleum Resources and Prospecting, Beijing, 102249 China; 3 CNPC Research Institute of Safety and Environmental Technology, Beijing, 102206 China

**Keywords:** Environmental sciences, Hydrology

## Abstract

Groundwater is unique resource for agriculture, domestic use, industry and environment in the Heihe River Basin, northwestern China. Numerical models are effective approaches to simulate and analyze the groundwater dynamics under changeable conditions and have been widely used all over the world. In this paper, the groundwater dynamics of the middle reaches of the Heihe River Basin was simulated using one numerical model and three machine learning algorithms (multi-layer perceptron (MLP); radial basis function network (RBF); support vector machine (SVM)). Historical groundwater levels and streamflow rates were used to calibrate/train and verify the different methods. The root mean square error and R^2^ were used to evaluate the accuracy of the simulation/training and verification results. The results showed that the accuracy of machine learning models was significantly better than that of numerical model in both stages. The SVM and RBF performed the best in training and verification stages, respectively. However, it should be noted that the generalization ability of numerical model is superior to the machine learning models because of the inclusion of physical mechanism. This study provides a feasible and accurate approach for simulating groundwater dynamics and a reference for model selection.

## Introduction

With the rapid development of information science and technology, groundwater models have been widely used in exploration of groundwater dynamics, quantitative assessment of groundwater resources^1,2^. A wide variety of models have been developed and applied for simulating groundwater dynamics which can be characterized as numerical (physical descriptive models) and empirical models. A major disadvantage of empirical models is the insufficient capability when confronting the dynamical behavior of the groundwater system changes. Many physically based numerical models for simulating groundwater system have been developed over the last 30 years^3–8^. Unfortunately, the numerical models have their own limitations such as requiring a large quantity of accurate data which can never be ascertained with absolute accuracy (e.g., the physical properties of aquifer). Furthermore, the computation resources can hardly satisfy the increasing refinement and complexity of numerical models. In recent years, machine learning methods (e.g., Artificial Neural Networks (ANNs)^9^, Support Vector Machine (SVM)^10^) have been used for forecasting in hydrologic research domains. Carlos *et al*. applied random forest algorithm to spatially predict the water retention of soils and achieved good performance on predicting volumetric water contents^11^. Gradient boosting^12^ is a dominant learning method for the Classification and Regression Tree (CART). Gradient Boosting Decision Tree (GBDT) has been successfully applied in various prediction problems^13^. Kenda *et al*. presented a research applying data-driven modeling methods (Regression Trees, Random Forests and Gradient Boosting) to predict groundwater level changes with sufficiently well performance using data collected in Ljubljana aquifer^14^. A model based on machine learning for predicting timely streamflow data was developed and tested in Idaho and Washington in four diverse watersheds with highly accurate and reliable predictions compared to the recorded data^15^. A method was proposed by combining Extreme Learning Machine and Quantum-Behaved Particle Swarm Optimization and assessed with daily runoff data of Xinfengjiang reservoir in China^16^. Worland *et al*. compared the ability of eight machine learning models and four baseline models to estimate the annual minimum 7-day mean streamflow in ungagged basins and concluded that machine learning methods can produce more accurate predictions in ungagged basins than baseline models^17^. Taormina *et al*. presented a research of applying Forward Neural Networks (FNNs) for long term simulations of groundwater levels in a coastal unconfined aquifer and suggested to regard FNNs as an alternative for numerical models^18^. The main advantage of this approach is that it does not require the complex nature of the underlying process of the physical systems as in numerical models.

Groundwater plays a significant role as sources of supply for domestic, industrial and agricultural purposes. Groundwater resources have been overexploited in many parts of the world^19^, especially in arid and semi-arid regions with highly variable precipitation and considerably high evapotranspiration. The depleted groundwater resources lead to environmental side effects including groundwater level declines, drying up of wells, increased pumping costs, land subsidence, decreased well yields, reduction of water in streams and lakes and water quality degradation^20,21^. Furthermore, population growth and climate extremes have significant influence on the quality and quantity of groundwater resources. Therefore, it is very important to sustainably manage groundwater resources in conjunction with surface water resources. Peng *et al*. analyzed the effects of water sources management strategies on water balance in North China and found reduced agriculture water consumption and sustained groundwater levels due to the decreased irrigation water use^22^. Sadeghi-Tabas *et al*. presented an attempt to link the multi-algorithm genetically adaptive search method (AMALGAM) with a numerical model to manage groundwater resources and found that “modeling - optimization - simulation” procedure was capable to obtain a set of optimal solutions^23^. For the effective management of groundwater resources, it is of great significance to simulate the groundwater dynamics accurately and reliably. Accurate assessments of groundwater levels allow water managers, engineers, and stakeholders to develop better strategies for groundwater management and balance the needs of urban, agricultural, industrial and other demands and analyze the benefits and costs of water conservation.

In this study, a physically based numerical model (MODFLOW, Modular Three-dimensional Finite-difference Ground-water Flow Model) and three machine learning methods were applied to simulate the groundwater dynamics of the middle reaches of Heihe River Basin, northwestern China. Collected data from 1986 to 2010 were divided into calibration/training and verification periods. The same data were used to calibrate/train different models. The objectives of our work are: (1) to explore the effectiveness of machine learning methods on simulating groundwater dynamics in arid basins; (2) to explore the applicability of machine learning methods and numerical models by comparing their results. The remainder of this paper is organized as follows: Section 2 presents methodologies for simulating the groundwater dynamics. Section 3 describes the study sites, the involved data and the processing of the data. The model structures, settings, hyperparameters and model performance criteria are presented in Section 4. Section 5 and 6 present the results, discussions and conclusions.

## Methods

### Multi-layer perceptron

ANNs are mathematical structure inspired by the biological neural networks proposed by McCulloch^24^. Multi-layer perceptron (MLP) is a class of feedforward ANN with input/output layers and several hidden layers. Nonlinear activation functions are used in the neurons to extract, learn and remember the nonlinear features and sub features from the inputs. Backpropagation is a family of methods which is always used to update the parameters in the ANN by calculating the gradient of a loss function with respect to all the parameters and back propagating the training errors^9,25^. Arbib summarized several researches which proved that any continuous functions can be approximated by feedforward neural network with one hidden layer^26^.

**Figure 1:**
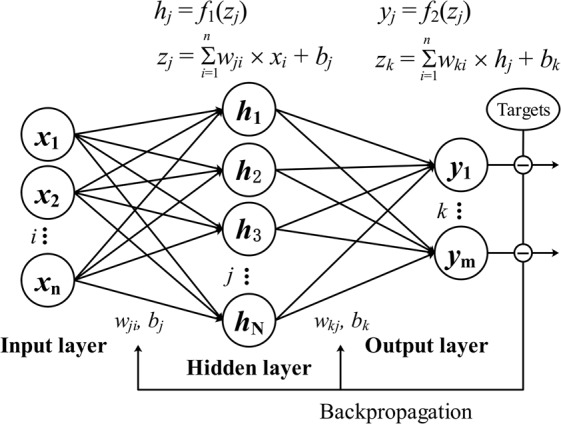
Schematic diagram demonstrating the architecture of backpropagation neural network. *x*_*i*_, *h*_*j*_ and *y*_*k*_ represent the nodal values in the input layer, hidden layer and output layer, respectively; *n*, *N* and *m* are the number of nodes in the input layer, hidden layer and output layer; *w*_*ji*_ is the weight connecting the input *x*_*i*_ and the *j*th neuron in the hidden layer; *w*_*kj*_ is the weight connecting the *j*th neuron in the hidden layer (*h*_*j*_) and the output *y*_*k*_; *b*_*j*_ and *b*_*k*_ are the biases in the hidden layer and output layer; *f*_1_ and *f*_2_ are the activation functions in the hidden layer and the output layer.

In this study, a feedforward MLP with one hidden layer was constructed and trained by backpropagation with gradient decent optimization algorithm (Fig. 1). The transfer function consists of a hyperbolic tangent sigmoid function in the hidden layer and a linear function in the output layer which is a most commonly used form. Detailed descriptions of MLP can be found in^27,28^.

### Radial basis function network

RBF network is generally a three-layer ANN using RBF as activation functions in the hidden layer. In the first layer (input layer), the number of neurons is identical to the input vectors. The radial basis functions in the hidden layer map the input vectors into a high dimension space. The neurons in the output layer of the network is calculated based on a linear combination of the hidden layer outputs (Eq. (1)). The characteristic feature of RBF is that the responses increase (or decrease) monotonically with Euclidean distance between the center and the input vectors. The architecture of RBF network is the same as MLP (shown in Fig. 1). Backpropagation is also used to update the parameters^29^.

1 $$f(x)=\mathop{\sum }\limits_{i=1}^{n}{w}_{i}\phi ({r}_{i},c)+{b}_{i}$$

Where, *n* is the number of nodes in the hidden layer; ${r}_{i}=\Vert x-{x}_{i}\Vert \,\,i=1,2,\mathrm{..}.,m$ is the Euclidean distance; *c* is non-negative prescribed parameter; *b* is bias; *Φ* represents RBF whose value depend only on the distance between the inputs and a fixed point^30^. Common used RBF include Gaussian, Multiquadric, Reciprocal Multiquadric, Thin-Plate Spline and Logistic^29^.

### Support vector machine

The SVM is proposed by Vapnik based on statistical learning theory^31^. SVM uses the concept of VC dimension and minimum structural risk to optimize and to obtain learning and generalization ability. Given by a set of *N* samples of ${\{{x}_{k},{y}_{k}\}}_{k=1}^{N},x\in {R}^{m},y\in R$ where *x* is an input vector of size *m* and *y* is the corresponding output value. An SVM estimator *f* on regression can be expressed as:

2 $$f(x)=w\cdot \phi (x)+b$$

Where *w* is a weight vector; *b* represents bias; *Ф* denotes a nonlinear transfer function which maps the input vectors into a high dimensional feature space. Vapnik^31^ introduced a convex optimization problem with an ε-insensitivity loss function to obtain the optimization for Eq. (2).

3 $$\mathop{\min }\limits_{w,b,\xi ,{\xi }^{\ast }}\,\,\frac{1}{2}{\Vert w\Vert }^{2}+C\mathop{\sum }\limits_{i=1}^{m}({\xi }_{i}+{\xi }_{i}^{\ast })$$

4 $$s.\,t.\{\begin{array}{c}w\cdot \phi ({x}_{i})+b-{y}_{i}\le \varepsilon +{\xi }_{i}\\ {y}_{i}-w\cdot \phi ({x}_{i})-b\le \varepsilon +{\xi }_{i}^{\ast }\\ {\xi }_{i},{\xi }_{i}^{\ast }\ge 0;\,i=1,2,\,\mathrm{..}.,\,m\end{array}\}$$

Where *ξ* and ξ^*^ are slack variables which involve “soft margin” to deal with infeasible constraints; *C* is a positive constant to penalize training errors by the loss function over the error tolerance *ε* and prevent overfitting. The optimization problem is usually solved by Duality Theory using Lagrangian multipliers and imposing Karush-Kuhn-Tucker (KKT) optimality condition. The structure of the estimator is supported by the input vectors which have nonzero Lagrangian multipliers under the KKT condition. Many algorithms have been proposed to solve the dual optimization problem of SVM^32,33^.

### Numerical model

In this study, MODFLOW^3^ is used to simulate the groundwater dynamics as a representation of numerical models for the purpose of comparison with machine learning methods. MODFLOW numerically solves the three-dimensional groundwater flow equation (Eq. (5)) using finite-difference method with determined initial and boundary conditions defined in Eqs. (6), (7), (8) and (9).

5 $$\frac{\partial }{\partial x}({K}_{xx}\frac{\partial h}{\partial x})+\frac{\partial }{\partial y}({K}_{yy}\frac{\partial h}{\partial y})+\frac{\partial }{\partial z}({K}_{zz}\frac{\partial h}{\partial z})-W={S}_{s}\frac{\partial h}{\partial t}$$

6 $${h(x,y,z,t)|}_{t=0}={h}_{0}\,\,\,\,x,y,z\in \varOmega ,\,t\ge 0$$

7 $${K}_{x}{(\frac{\partial h}{\partial x})}^{2}+{K}_{y}{(\frac{\partial h}{\partial y})}^{2}+{K}_{z}{(\frac{\partial h}{\partial z})}^{2}-\frac{\partial h}{\partial z}({K}_{z}+p)+p=\mu \frac{\partial h}{\partial t}\,\,\,\,x,y,z\in {\varGamma }_{0}$$

8 $${h(x,y,z,t)|}_{{\varGamma }_{1}=0}={h}_{1}(x,y,z)\,\,\,\,x,y,z\in {\varGamma }_{1},\,t\ge 0$$

9 $$-{K}_{n}{\frac{\partial h}{\partial n}|}_{{\varGamma }_{2}}=q(x,y,z,t)\,\,\,\,x,y,z\in {\varGamma }_{2},\,t\ge 0$$

Where *K*_*x*_, *K*_*y*_ and *K*_*z*_ are values of hydraulic conductivity along the *x*, *y*, and *z* coordinate axes (L•T^−1^); *h* is the hydraulic head (L) which can be converted to groundwater level; *W* represents source and/or sink term of water (1/T) with *W*<0.0 for flowing out of the groundwater system, and *W*>0.0 for flowing into the system; *S*_*s*_ denotes the specific storage of the aquifer (1/L); *t* is time (T); *h*_0_ is the initial hydraulic head (L); *Ω* denotes the study area; *n* is normal direction of a hydraulic boundary; *Г*_1_ denotes the top boundary condition of the study area; *Г*_1_ and *Г*_2_ are the Dirichlet boundary condition and Neumann boundary condition; and *q*(*x*, *y*, *z*, *t*) is the normal discharge per unit width (L^2^(d•L)^−1^). Solution of the groundwater flow equation is achieved by finite-difference method in which the groundwater flow system and simulation time are discretized into grids and stress periods, respectively. Each stress period is a period of simulation within which specified stress data are constant.

## Study sites and data descriptions

### Study sites

**Figure 2:**
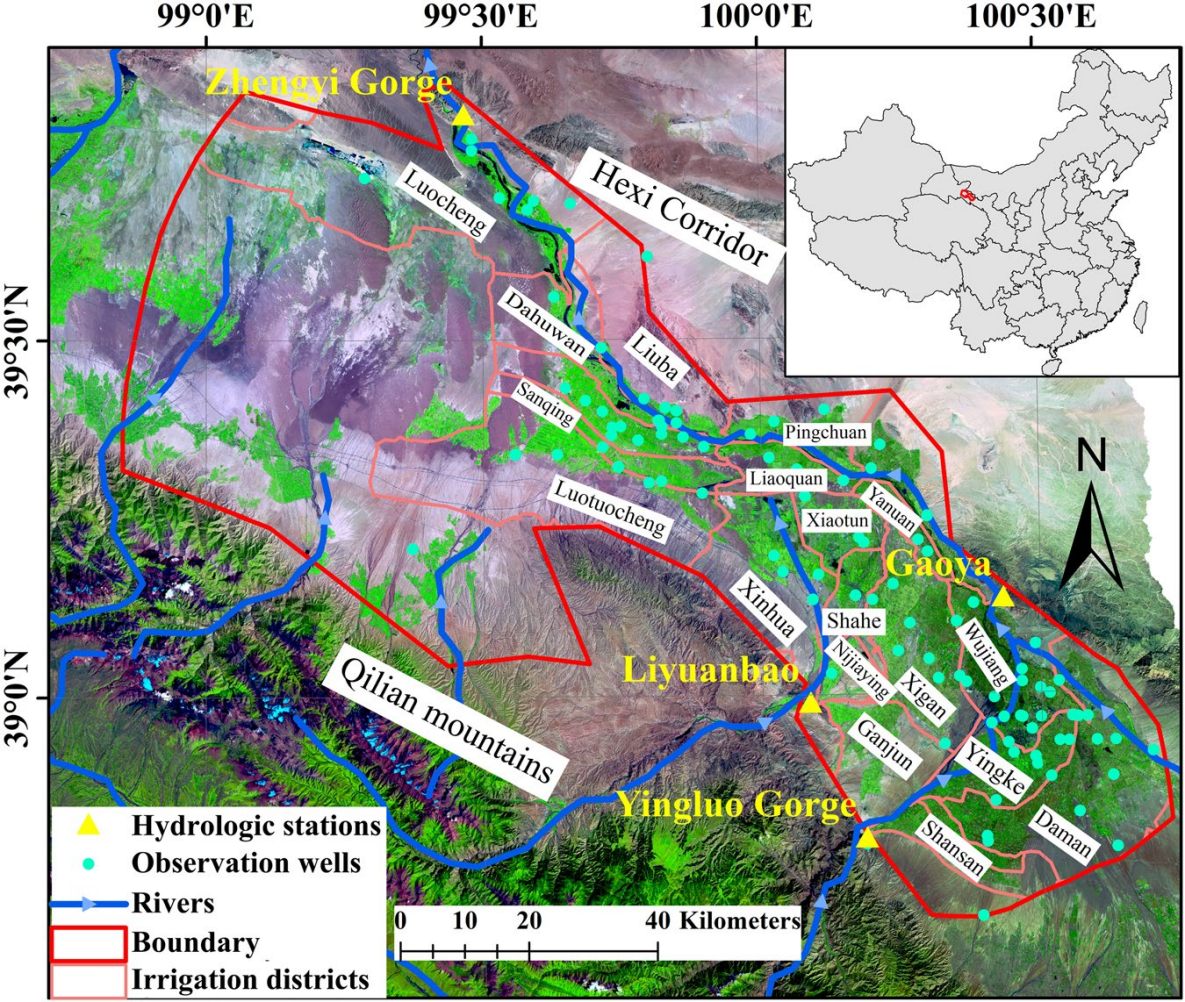
Map of the middle reaches of the Heihe River Basin. (Note: the map was generated using ESRI’s ArcGIS 10.2 (http://desktop.arcgis.com/en/arcmap/); the satellite imagery was provided by Cold and Arid Regions Sciences Data Center at Lanzhou (http://westdc.westgis.ac.cn).

The Heihe River Basin which located in the middle of Qilian Mountain is the second largest inland river basin in the northwest of China. The basin extends ~821 km with an area of ~14 × 10^4^ km^2^. The middle reaches of the Heihe River Basin (38 °38′N-39°53′, 98 °53′E-100°44′E; Fig. 2) with an area of ~9016 km^2^ was selected as the study area. The groundwater resource in this area has been overexploited for agricultural, industrial, and domestic use. The water system of the Heihe River Basin is composed of 35 independent rivers among which most of the mountainous rivers dry up because of irrigation water withdrawal and recharging to the aquifer in front of the mountains. The major rivers in the study area are the mainstream of the Heihe River and the Liyuan River. The Heihe River flows in the study area through the Yingluo Gorge hydrologic station and flows out of the study area through the Zhengyi Gorge hydrologic station (Fig. 2).

### Data

Various kinds of data including Digital Elevation Model (DEM), land use data, groundwater pumping yields, groundwater levels, streamflow rates, etc., were used in this study. All the available data were used to construct the numerical model; however, only time-variant data (i.e., streamflow rates, groundwater pumping rates, agricultural irrigation, and groundwater levels) were used to establish the machine learning models. Land use data were obtained through visual interpretation of Landsat TM/ETM+ images in 1986^34^, 2000^35^ and 2007^36^. Historical data of groundwater levels from 42 monitoring wells (light blue dots in Fig. 2) were collected by the Gansu Provincial Bureau of Hydrology and were used in the study. The irrigation data were obtained from annual water resource management reports published by the Zhangye Municipal Bureau of Water Conservancy. Annual runoff at Yingluo, Gaoya and Zhengyi hydrologic stations (yellow triangle in Fig. 2) were collected from the Gansu Provincial Bureau of Hydrology. The data of groundwater exploitation during the modeling period were obtained from China Census for Water. All the above-mentioned data were obtained from the “China Western Environment and Ecology Science Data Center” (http://westdc.westgis.ac.cn).

### Data processing

Elevation, irrigation, streamflow rates and pumping yields were processed to drive the numerical model. The elevation of the surface and bottom of the study area was obtained from the DEM which provided by the CGIAR-CSI GeoPortal. The resolution of the elevation was processed to 1 km from 90 m. Time-variant data were transformed into monthly stress periods (time interval) from January 1986 to December 2010. The calibration and verification periods were chosen as 1986–2008 and 2009–2010 because of the availability of relatively complete historical records. The main channels, tributaries and the divisions of the Heihe River were implemented using the Streamflow-Routing (STR) package^37^. The streamflow rates measured at the Yingluo Gorge hydraulic station and Liyuan River were assigned to the STR package to simulate the rivers. Basic parameters (Stream state, top elevation of the streambed, bottom elevation of the streambed, width of the stream channel) were derived from^38^. The agricultural irrigation was implemented using Recharge (RCH) package^3^ which combined the surface water and groundwater irrigation. The groundwater exploitation was simulated using the Well package^3^ by assigning pumping rates which were calculated from the extraction records.

Only time-variant data including streamflow rates, groundwater pumping rates, agricultural irrigation, and groundwater levels were used to construct the machine learning models. The time-series dataset was divided into two parts in accordance with the two stages in the numerical model building process: training and testing. The training and testing periods were 1986–2008 and 2009–2010, respectively. The input and output data were summarized in Table 1 from which we could find existence of different units and ranges which would have influence on the results. Therefore, a normalization procedure was conducted for the machine learning methods to nondimensionalize the data to eliminate the effects of dimension as shown in Eq. (10). The data were normalized to the range of (−1, 1) after the procedure.

10 $${x}^{\ast }=\frac{({y}_{\max }-{y}_{\min })\times (x-{x}_{\min })}{{x}_{\max }-{x}_{\min }}+{y}_{\min }$$

Where *x* is the original data; x^*^ represents the data after nondimensionalizing; *x*_*min*_ and *x*_*max*_ are the minimum and maximum value of *x*; *y*_*min*_ and *y*_*max*_ are the lower and upper bound of the normalized data.

**Table 1:** Input and output data for machine learning models.

		Unit	Range
Inputs	Pumping rates	(m^3^/day)	(−1 × 10^4^, 0)
	Recharge rates	(m/day)	(0, 1 × 10^–2^)
	Streamflow rates at Yingluo Hydrologic station	(m^3^/day)	(7 × 10^5^, 2 × 10^7^)
	Streamflow rates of Liyuan River	(m^3^/day)	(0, 4 × 10^6^)
Outputs	Groundwater levels	(m.a.s.l)	(1 × 10^3^, 1.5 × 10^3^)
	Streamflow rates at Gaoya Hydrologic station	(m^3^/day)	(2 × 10^4^, 1.5 × 10^7^)
	Streamflow rates at Zhengyi Gorge Hydrologic station	(m^3^/day)	(0, 1.5 × 10^7^)

## Model development

### Numerical model settings

**Figure 3:**
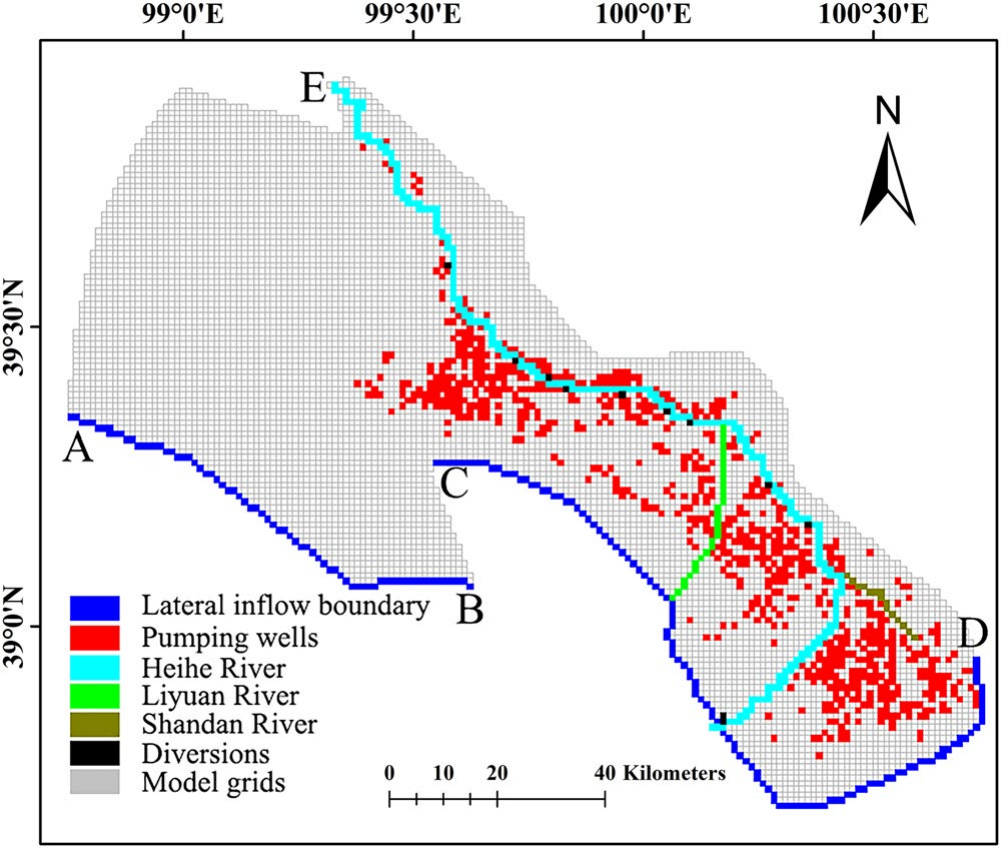
The numerical discretization and boundary conditions for the middle reaches of the Heihe River Basin.

The study area was numerically discretized by quadrate grids. The finite-difference grid consisted of 132 rows and 160 columns with a uniform cell size 1 × 1 km (Fig. 3). Only one layer was simulated with the surface and bottom elevation deriving from^39,40^. The initial hydraulic heads were determined using the monitoring groundwater level data in 89 groundwater wells observed in January 1986 and were spatially interpolated by applying Kriging interpolation method in ArcGIS.

Groundwater flow in the aquifer is governed by the boundary conditions. The lateral hydraulic boundaries of the study area were coincided with earlier studies^39,41,42^ and defined by the natural boundaries (Fig. 3). A no-flow boundary was defined between A-E, because a groundwater divide was present at this boundary. E was the outlet of HeiHe River in the middle reach which was coincident with the Zhengyi Gorge reservoir. The groundwater flow from the mountain to the model domain through D-E cannot be exactly quantified. No-flow boundary was defined between D-E as the hydraulic conductivity in the hard rock was significantly smaller than that of the basin sediments according to a previous study^43^. The most complicated hydraulic boundary was the south boundary (between A and D) in the study domain. Because of various lateral inflows, including several gully flows, deep lateral seepage from mountains and the Heihe River inflow, the boundaries were separated into several sections according to the hydraulic conditions along the boundary. As shown in Fig. 3, constant flux cells were defined between A-B and C-D where groundwater flows into the model domain from mountains and the fixed-flow boundary was realized using Well Package in MODFLOW; no-flow boundary were specified between B-C. The top boundary was atmospheric air-soil interface. The bottom boundary condition at the base of aquifer was defined to no-flow boundary. The discretization of the groundwater system is shown in Fig. 3.

### Development of machine learning methods

All the machine learning methods were carried out in MATLAB 2017a environment running on a Intel Core i5, 2.5 GHZ CPU with DDR3L, 1600MHz RAM. The number of input layer neurons and output layer neurons were set based on the dimension of the input data and output data. The dimensions of input data include pumping rates and recharge rates of 21 irrigation districts (light red polygon in Fig. 2) and streamflow rates of two rivers (blue polyline in Fig. 2). The dimensions of the output data include groundwater levels observed at 42 boreholes (light blue dots in Fig. 2) and streamflow rates from two hydrologic stations (yellow triangle in Fig. 2). Therefore, the number of neurons in the input layer and output layer were both 44. As for the MLP, the hyperbolic tangent sigmoid transfer function and linear transfer function were applied in the neurons of the hidden layer and output layer, respectively. The number of hidden neurons was identified by trial and error procedure which started with two hidden neurons initially and increased to 10 with a step size of 1 at each trial. For each set of hidden neurons, the network was trained to minimize the Mean Square Error (MSE) at the output layer. Levenberg-Marquardt algorithm was used to update the values of weights and biases. The training was stopped when there was no significant improvement in the performance. The parsimonious structure that resulted in minimum error and maximum efficiency during training was selected as the final form of MLP. As for the RBF network, the Gaussian radial basis function and linear transfer function were applied in the neurons of the hidden layer and output layer, respectively. The number of hidden neurons was also identified by trial and error procedure which started with two hidden neurons and increased to 70. For each set of hidden neurons, the worst performing vector is added to the hidden layer as a Gaussian transfer function center to improve performance. Then the linear transfer function in the output layer was readjusted to minimize the MSE. As for the SVM, Gaussian function (also called radial basis function) was used as kernel function to compute the Gram matrix. Sequential minimal optimization (SMO)^44^ was used to solve Eqs. (3) and (4). The output of SVM regression predictor was a one-dimensional vector. Therefore, 44 SVM regression models were trained using all 44-input data for each output vector. After training the machine learning methods, the machine learning models (MLP model, RBF model and SVM model) were generated for the study area.

### Performance criteria

As recommended by^45^, the Root Mean Square Error (RMSE) and Coefficient of Determination (R^2^) were used as objective functions to assess the groundwater level simulations through the calibration (training), verification (testing) stages (as shown in Eqs. (11) and (12)). The RMSE measures the average magnitude of the error between model simulations (*M*) and observations (*O*). As shown in Eq. (13), the errors are squared before averaged, large errors take a relatively high weight. Therefore, RMSE is useful when large errors are undesirable and R^2^ measures the predictive ability of models.

11 $$RMSE=\sqrt{\frac{1}{N}\mathop{\sum }\limits_{i=1}^{N}{({M}_{i}-{O}_{i})}^{2}}$$

12 $${R}^{2}=1-\frac{\mathop{\sum }\limits_{i=1}^{N}{({O}_{i}-{M}_{i})}^{2}}{\mathop{\sum }\limits_{i=1}^{N}{({O}_{i}-\overline{O})}^{2}}$$

Where *N* represents the total number of observations; $\overline{O}$ is the average of observations.

In the development of data-driven models (e.g., MLP, RBF, SVM), the most important issue is to guarantee the generalization ability of the models. Therefore, the generalization ability (GA) is evaluated as follows:^46^

13 $$GA=\frac{RMSE\,in\,prediction\,stage}{RMSE\,in\,training\,stage}$$

The *GA* values are unity if the models simulate the groundwater system perfectly. However, if the models are over calibrated/trained, the *GA* values exceed unity. *GA* values less than unity indicates that the model is under calibrated/trained.

Besides, the elapsed time in data preparation, calibration and computation should be recorded as a criterion to assess different models. However, the elapsed time in data preparation process was not considered because of the same input and output data in different models. Therefore, the elapsed time in calibration and computation is considered in this study.

## Results and discussions

A physically based numerical model (MODFLOW) and three machine learning methods (MLP, RBF and SVM) were applied to construct the groundwater models. The models were calibrated/trained and verified using two datasets of observed groundwater level and streamflow rates. The results of calibration/training, verification and generalization ability from each model were demonstrate in this section. The RMSE and R^2^ were used to evaluate the results. Furthermore, the comparisons between different models were conducted to explore the applicability of machine learning methods and numerical models.

### Model calibration/Training

**Figure 4:**
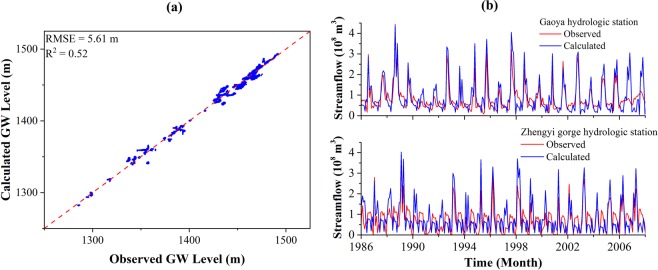
(**a**) Comparison of the observed and simulated groundwater level in calibration period. Blue dots refer to the scatter plot of the observed and simulated groundwater level, the red dashed line denotes a perfect match where “simulated groundwater level = observed groundwater level”; (**b**) Comparison of the observed and simulated streamflow rates at Gaoya (*upper*) and Zhengyi (*lower*) Gorge hydraulic stations in calibration period. The blue curve refers to the simulated streamflow rates, the red curve denotes the observed streamflow rates.

The numerical model was calibrated from January 1986 to December 2008 with monthly stress periods. The hydraulic parameters and hydraulic boundary conditions were calibrated using two types of data, consisting of observed groundwater level from 42 boreholes and streamflow rates measured at Gaoya and Zhengyi Gorge hydrologic stations. The calibration makes the simulated results match the observed groundwater level data from the monitoring wells as much as possible. The observed and simulated groundwater level at all the observation wells (42 boreholes) is shown in Fig. 4(a) with the RMSE value of 5.61 m and R^2^ value of 0.52. Figure 4(b) shows the comparison between the observed and simulated monthly streamflow rates at Gaoya and Zhengyi Gorge hydrologic stations with RMSE value of 1.76 × 10^6^ m^3^/day and R^2^ value of 0.51. The results indicated a reasonable match for the numerical model in the calibration period.

**Figure 5:**
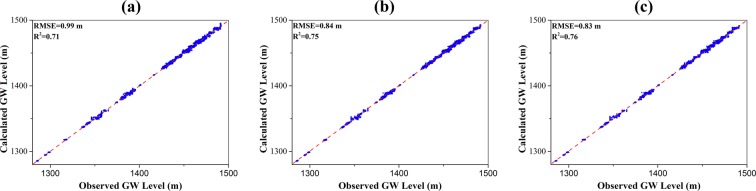
Comparison of the observed and simulated groundwater level for (**a**) MLP model; (**b**) RBF model; and (**c**) SVM model. Blue dots refer to the scatter plot of the observed and simulated groundwater level, the red dashed line denotes a perfect match where “simulated groundwater level = observed groundwater level”.

**Figure 6:**
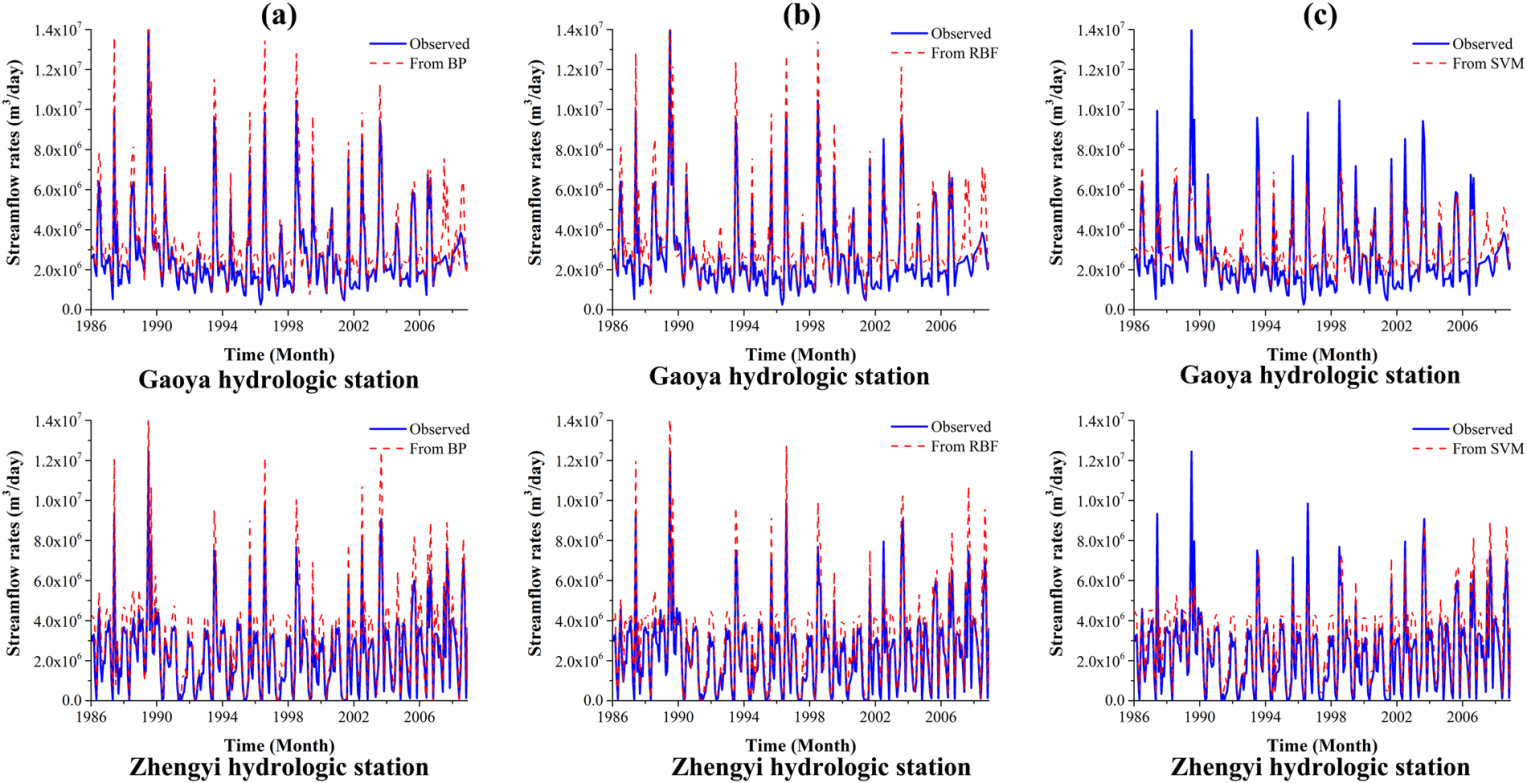
Comparison of the observed and simulated streamflow rates at Gaoya (*upper*) and Zhengyi (*lower*) Gorge hydraulic stations for (**a**) MLP model; (**b**) RBF model; and (**c**) SVM model. The blue curve refers to the simulated streamflow rates, the red dashed curve denotes the observed streamflow rates.

The monthly data from 1986 to 2008 was used to train the MLP, RBF network, and SVM. The trained groundwater levels from machine learning models are shown in Fig. 5. In the training stage, the RMSE and R^2^ values for MLP, RBF network, and SVM models are 0.99 m and 0.71, 0.84 m and 0.75, 0.83 m and 0.76. The results from SVM model are slightly better than those of MLP and RBF models. The results from MLP model is the worst with RMSE value of 0.99 m and R^2^ value of 0.71. The RMSE discrepancy is reasonable considering the relatively large difference between the highest and lowest groundwater level with about 230 m. The RMSE and R^2^ value for MLP, RBF, and SVM models are 1.09 × 10^6^ m^3^/day and 0.66, 1.16 × 10^6^ m^3^/day and 0.66, 1.16 × 10^6^ m^3^/day and 0.66 at Gaoya hydrologic station and Zhengyi Gorge hydrologic station (Fig. 6). According to^45^, the results could be considered acceptable when R^2^ values greater than 0.5. The results indicate that the machine learning methods are reasonable for simulating (learning) the groundwater dynamics for the middle reaches of the Heihe River Basin.

### Verification

**Figure 7:**
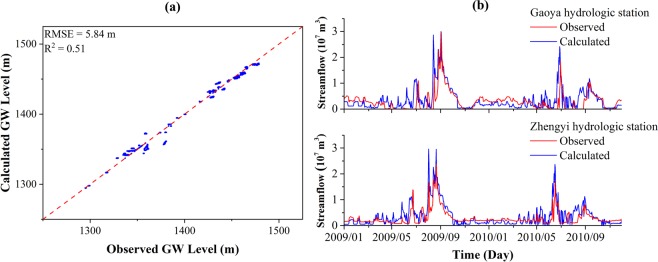
(**a**) Comparison of the observed and simulated groundwater level in verification period. Blue dots refer to the scatter plot of the observed and simulated groundwater level, the red dashed line denotes a perfect match where “simulated groundwater level = observed groundwater level”; (**b**) Comparison of the observed and simulated streamflow rates at Gaoya (*upper*) and Zhengyi (*lower*) Gorge hydraulic stations in verification period.

The calibrated numerical model was verified using the data of 2009 and 2010. The stress period and the hydraulic parameters and boundary conditions were identical to the calibration period. The calculated groundwater level of the last time step from calibration period were used as the initial heads of the verification period. The comparison between observed and simulated groundwater levels and streamflow rates are shown in Fig. 7(a,b), respectively. The RMSE value and R^2^ value for the groundwater levels are 5.84 m and 0.51, respectively. The calculated streamflow rates of Gaoya and Zhengyi Gorge hydrologic stations shown in Fig. 7(b) match the observed streamflow rates considerably. Inspection of the comparison between calculated and observed groundwater levels and streamflow rates during the calibration and verification periods elucidates that the assumptions of boundary conditions made for the study area are appropriate and the establishment of the groundwater model for the middle reaches of the Heihe River Basin is feasible.

**Figure 8:**
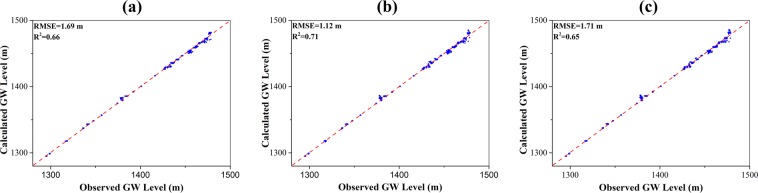
Comparison of the observed and simulated groundwater levels for (**a**) MLP model; (**b**) RBF model; (**c**) SVM model. Blue dots refer to the scatter plot of the observed and simulated groundwater level, the red dashed line denotes a perfect match where “simulated groundwater level = observed groundwater level”.

**Figure 9:**
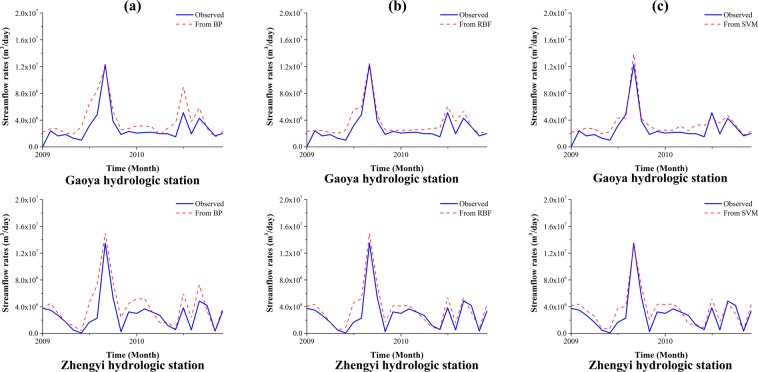
Comparison of the observed and simulated streamflow rates at Gaoya (*upper*) and Zhengyi Gorge (*lower*) hydraulic stations for (**a**) MLP model; (**b**) RBF model; (**c**) SVM model. The blue curve refers to the simulated streamflow rates, the red dashed curve denotes the observed streamflow rates.

Figure 8 shows the comparison the observed and simulated groundwater levels for machine learning models in verification period. The models trained in the training stage were used to predict by applying new input data. The RMSE and R^2^ values were calculated using the model outputs and new observations. The RMSE and R^2^ values are 1.69 m and 0.66, 1.12 m and 0.71, 1.71 m and 0.65 for MLP, RBF, and SVM models, respectively. The streamflow rates predicted by machine learning models are shown in Fig. 9. The RMSE value and R^2^ value for MLP, RBF, and SVM models calculated from streamflow rates at Gaoya and Zhengyi Gorge hydrologic stations are 1.69 × 10^6^ m^3^/day and 0.54, 1.21 × 10^6^ m^3^/day and 0.79, 1.17 × 10^6^ m^3^/day and 0.83. In the verification period, the model based on RBF network performs the best. This may due to the local transfer function and relatively large number of neurons in the hidden layer. The ANN methods (MLP and RBF network) are always based on an assumption of unlimited samples which can never be satisfied. The origin of SVM is based on limited samples and follows the structural risk minimization which adequately balanced the accuracy and generalization ability. SVM maps the input vectors into high-dimensional feature space by support vector and manage the problem following the linear optimization algorithm which avoids local minimum and Curse of Dimensionality.

### Generalization ability

The generalization ability was evaluated by Eq. (13) which indicates that *GA* values are greater if the model concentrates on learning the given training data rather than a more general system and that the higher the index values are, the weaker the generalization ability becomes. *GA* values (Table 2) calculated from groundwater level for MLP, RBF, and SVM models are 1.7, 1.3, and 2.1 which implies that the generalization ability of the RBF model is superior to that of MLP and SVM models. *GA* values calculated from streamflow rates for MLP, RBF, and SVM models are 1.55, 1.04, and 1.00. The overall values of *GA* which averages the two values of indices are 1.63, 1.18, and 1.53 which indicates that the generalization ability of RBF model is the lowest. Similar to the machine learning models, the generalization ability of numerical model was also evaluated by calculating *GA* values. The GA values calculated from groundwater level and streamflow rates for numerical model are 1.04 and 1.11 with the average of 1.08.

**Table 2:** Comparison of generalization ability.

	Numerical model	MLP model	RBF model	SVM model
Groundwater level	1.04	1.70	1.33	2.06
Streamflow rates	1.11	1.55	1.04	1.00
Overall	1.08	1.63	1.18	1.53

### Comparisons

**Figure 10:**
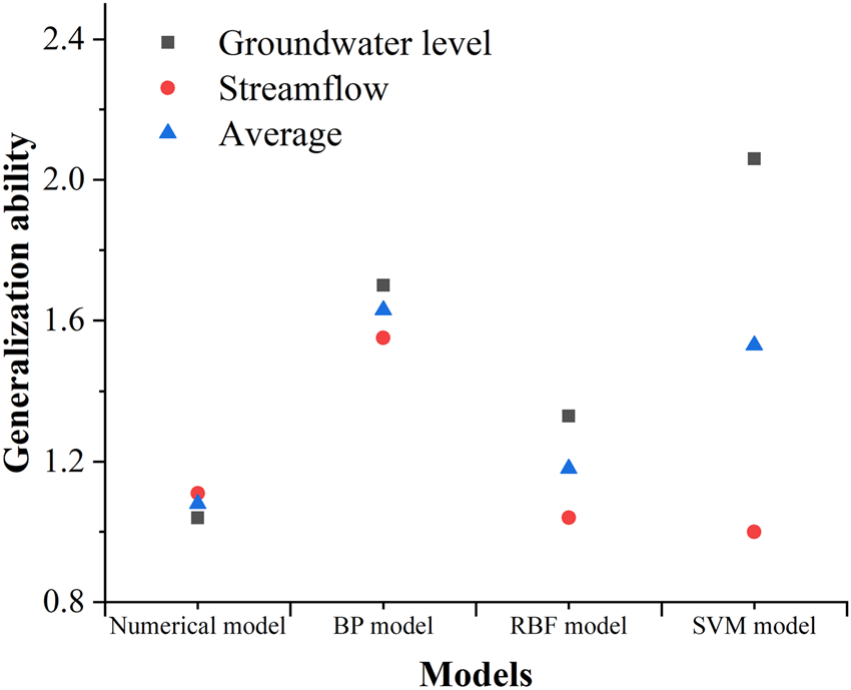
The comparison of generalization ability between different models.

The comparison of numerical model and machine learning models in the calibration/training stage was conducted and shown in Table 3. RMSE and R^2^ values were used to evaluate the accuracy of the simulated groundwater levels and streamflow rates compared to the observations. In this study, the RMSE and R^2^ values imply that the accuracy of machine learning models is better than that of numerical model for the given data. Furthermore, the time elapsed in constructing the model is divided into two parts which are calibration/training time and computation time. The calibration of numerical model usually costs the hydrologist months to balance lots of aspects, processes and parameters. However, the machine learning methods only cost experts’ days to determine the hyperparameters after data preparation. This is also the main reason why the calibration of the models is described in detail. Among the machine learning methods, the reproduction capability of groundwater levels and streamflow rates of RBF network and SVM is superior to that of MLP which may be caused by different transfer functions, network structures, and minimizing methods. The comparison between numerical model and machine learning methods in the verification/prediction stage is shown in Table 4. The performance of RBF model is better than that of numerical model, MLP model, and SVM model which indicates that RBF network is applicable to simulate groundwater systems. The comparison of generalization ability between different models is shown in Fig. 10. The generalization ability of numerical model calculated from groundwater levels is better than those of machine learning methods. The generalization ability of SVM model calculated from streamflow rates performs the best among the all the models. It is noted that the overall generalization ability of the numerical model is superior to those of machine learning methods with lower generalization ability index value. The relatively less difference of generalization ability calculated from groundwater levels and streamflow rates indicates the stability of the numerical models. On the one hand, the RMSE value in calibration stage of numerical model which act as denominator in Eq. (13) is relatively large. On the other hand, the dynamics simulated by numerical model are based on the groundwater flow equation (Eq. (5)) with the same boundary conditions and parameters which dominates the groundwater movements. On the contrary, the machine learning methods are mappings between the inputs and outputs based on statistics without deduction of physical process. In the machine learning methods, the RBF model performs the best in generalization ability which is also close to the numerical model.

**Table 3:** Comparison in the calibration/training stage.

		Numerical model	MLP model	RBF model	SVM model
RMSE	Groundwater level (m)	5.61	0.99	0.84	0.83
	Streamflow rates (m^3^)	1.76 × 10^6^	1.09 × 10^6^	1.16 × 10^6^	1.16 × 10^6^
R^2^	Groundwater level	0.52	0.71	0.75	0.76
	Streamflow rates	0.51	0.66	0.66	0.66
Time	Calibration	months	days	days	days
	Computation	1898 s	716.9 s	4.2 s	1.0 s

**Table 4:** Comparison in the verification stage.

		Numerical model	MLP model	RBF model	SVM model
RMSE	Groundwater level (m)	5.84	1.69	1.12	1.71
	Streamflow rates (m^3^)	2.05 × 10^6^	1.69 × 10^6^	1.21 × 10^6^	1.17 × 10^6^
R^2^	Groundwater level	0.51	0.66	0.71	0.65
	Streamflow rates	0.50	0.54	0.79	0.83
	Time (s)	30	0.07	0.06	0.10

## Conclusions

In this paper, the groundwater dynamics in the middle reaches of Heihe River Basin were simulated by numerical models and machine learning methods. Historical data of groundwater levels and streamflow rates were used to calibrate/train and verify/test the models. The RMSE and R^2^ values were used to evaluate the simulated results of the constructed model which indicated that the calibrated model could considerably reproduce the trend and values of historical observations. Furthermore, a comparison was conducted to discover pros and cons of different models. The results showed that the performances of machine learning models on simulating historical data was superior to those of numerical model with RBF model performed the best. The computation cost of machine learning models in training and prediction stages were much less than those of numerical model in calibration and verification stages. However, the generalization ability of the numerical model was better than that of machine learning methods because of the physical based mechanism. Therefore, machine learning models are applicable to the scenarios which require numerous executions without considering the physical mechanisms (e.g., real-time models, sensitivity/uncertainty analysis, and optimizations). The developed models and the results of this study may be useful for the accurate groundwater management, decision making, and model selection. Future research should be focused on exploring applicability of deep learning methods or tree-based machine learning algorithms in hydrologic field and application of the developed models to manage groundwater resources.
